# (Lack of) Effects of noradrenergic stimulation on human working memory performance

**DOI:** 10.1007/s00213-020-05590-0

**Published:** 2020-06-25

**Authors:** Nadine Wanke, Jana Christina Müller, Klaus Wiedemann, Lars Schwabe

**Affiliations:** 1 Department of Cognitive Psychology, University of Hamburg, 20146 Hamburg, Germany; 2 Department of Psychiatry, University Clinic Hamburg-Eppendorf, Hamburg, Germany

**Keywords:** Yohimbine, Noradrenaline, Norepinephrine, Working memory, *n*-back, Prefrontal cortex, Learned helplessness

## Abstract

**Rationale:**

Working memory depends on prefrontal cortex functioning, which is particularly sensitive to levels of noradrenaline. Studies in non-human primates have shown that modest levels of noradrenaline improve working memory, and that higher levels of noradrenaline impair working memory performance. However, research in humans provided inconsistent findings concerning noradrenergic effects on working memory.

**Objective:**

The present study aimed at assessing dose-dependent effects of yohimbine, an alpha-2 adrenoceptor antagonist, on working memory performance in healthy humans. We further aimed to explore a potential interactive effect between noradrenergic arousal and lack of control over aversive events on working memory performance.

**Methods:**

We used a double-blind, fully crossed, placebo-controlled, between-subject design. Participants (*N* = 121) performed an adaptive *n*-back task before and after oral administration of either a placebo, 20 mg, or 40 mg yohimbine and a manipulation of controllability, during which participants could either learn to avoid electric shocks (controllability groups), had no instrumental control over shock administration (uncontrollability groups), or did not receive any shocks (no-shock control group).

**Results:**

While no significant results of noradrenergic stimulation through yohimbine were obtained using conventional frequentist analyses, additional Bayesian analyses provided strong evidence for the absence of an association between pharmacological treatment and working memory performance. We further observed no effect of controllability and no interaction between noradrenergic stimulation and the manipulation of controllability.

**Conclusions:**

Our results suggest that noradrenergic stimulation through yohimbine does not affect (non-spatial) working memory in healthy human participants.

## Introduction

Working memory is a core cognitive function that is essential for goal-directed behavior. At a neural level, working memory relies heavily on the dorsolateral prefrontal cortex (Barbey et al. 2013; Cohen et al. 1997; D'Esposito and Postle 1999). This brain area is known to be regulated by catecholamines in general and noradrenaline in particular (Arnsten 2011; Robbins 2000). Increased noradrenergic arousal, mediated by noradrenergic projections from the locus coeruleus to prefrontal areas, is assumed to be a driving force in the disruptive effect of stressful events on working memory function (Bogdanov and Schwabe 2016; Schoofs et al. 2009). Noradrenergic arousal-related changes in working memory are further thought to be relevant in mental disorders, such as ADHD (Vanicek et al. 2014) and depression (Gartner et al. 2018), and might thus point to potential treatment approaches for these disorders.

The most compelling evidence for an association between noradrenaline and working memory comes from animal studies. These studies suggest that noradrenaline levels both below or above an optimal level impair working memory performance (Arnsten 2011; Ramos and Arnsten 2007; Ramos et al. 2005). Evidence from non-human primates suggested further differential effects of alpha-2(A) adrenergic and alpha-1 adrenergic receptors in the impact of noradrenaline on working memory performance. While alpha-2 adrenergic receptors are assumed to engage at moderate levels of noradrenaline and to lead to enhanced performance (Franowicz et al. 2002), alpha-1 adrenergic receptors are thought to be engaged at higher levels of noradrenaline and to impair working memory performance (Birnbaum et al. 1999). To what extent these findings can be translated to humans, however, is unclear. Empirical evidence for a relationship between noradrenaline and working memory functioning in humans is mixed, especially with regard to pharmacological agents modulating alpha adrenergic receptors (Chamberlain et al. 2006; Chamberlain and Robbins 2013). The alpha-2 adrenoceptor agonist clonidine has been shown to impair working memory (Smith et al. 2003; Tiplady et al. 2005), which is difficult to interpret due to its parallel effects on attention and arousal (Chamberlain and Robbins 2013; Clark et al. 1986; Coull et al. 1995; Coull et al. 2001; Jakala et al. 1999). However, for other pharmacological agents targeting alpha adrenergic receptors, such as desipramine, guanfacine, or yohimbine, there was no tendency for or against an impairment of working memory (Chamberlain et al. 2006; Chamberlain and Robbins 2013). These inconclusive findings may be due to differences in drug dosage (Chamberlain et al. 2006), sample characteristics (Muller et al. 2005), or task difficulty (Campbell et al. 2008).

Both the release of noradrenaline and impaired prefrontal functioning have further been associated with exposure to uncontrollable stressors (Arnsten 2009; Minor et al. 1984). Early research in rodents showed that lack of control over aversive events compared with the same events with instrumental control led to escape deficits later on, as reflected in reduced motor activity in a swim test (Weiss et al. 1981), a phenomenon that had been observed in dogs before (Overmier and Seligman 1967; Seligman and Maier 1967). It was further shown that these deficits were paralleled by a depletion of noradrenaline in the locus coeruleus (Weiss et al. 1981). Notably, depletion of noradrenaline in the locus coeruleus actually increases noradrenergic activity in cortical areas through inhibitory autoreceptors on locus coeruleus neurons (Samuels and Szabadi 2008; Weiss and Simson 1988). Studies in humans indicated that uncontrollable stressors activate central stress systems (Breier et al. 1987), as indicated by higher plasma levels of adrenocorticotropic hormone and noradrenaline after exposure to uncontrollable as opposed to controllable stress, but did not disentangle the effects of noradrenaline from those of other stress mediators, such as cortisol. Accordingly, the role of noradrenaline in cognitive deficits after exposure to uncontrollable stressors in humans is currently unknown.

**Fig. 1:**
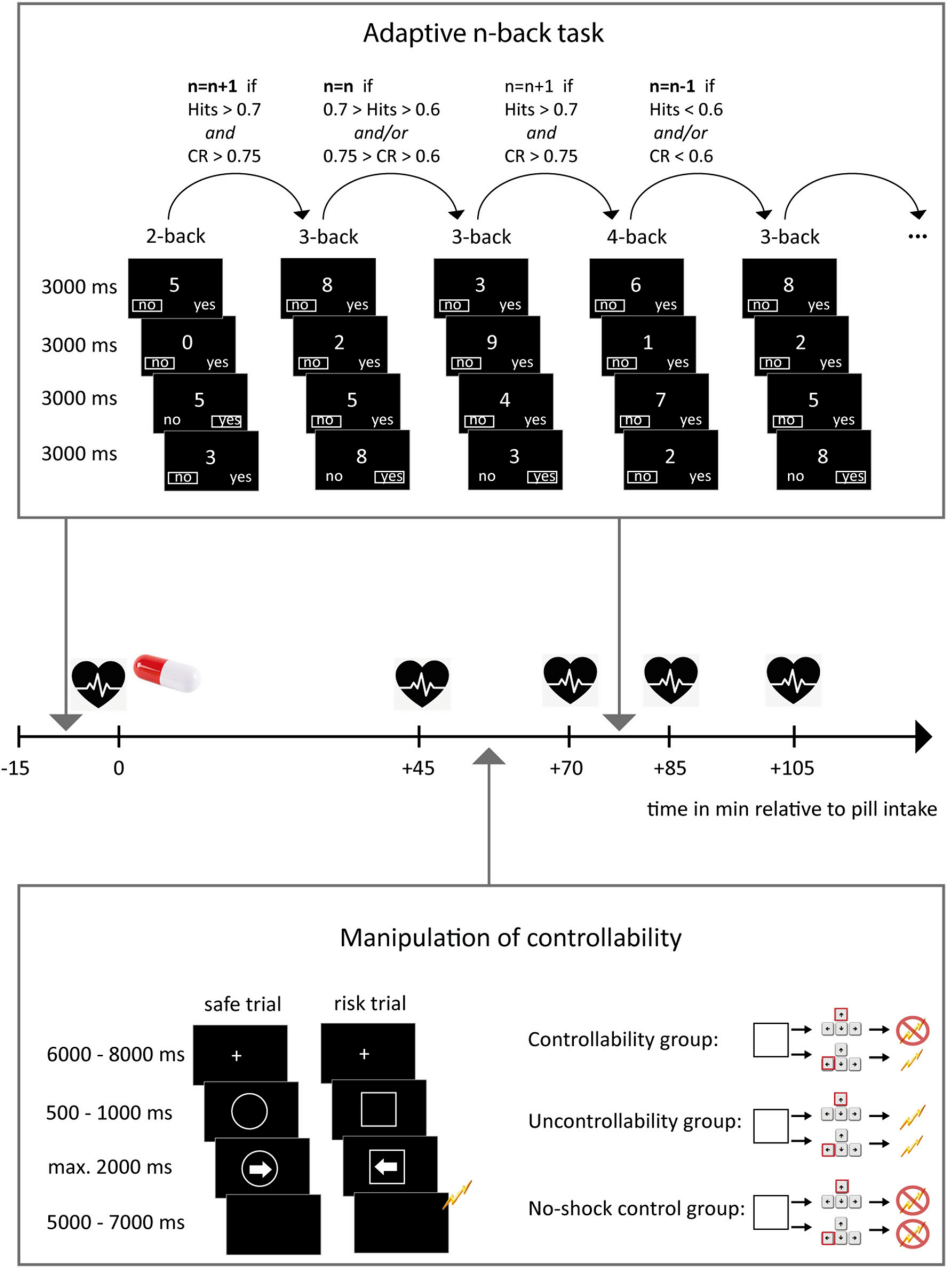
Experimental design and procedure. Participants performed a baseline session of an adaptive *n*-back task in which they were requested to indicate for each number presented on the computer screen whether the number was different from the number that had been displayed *n* positions before. The task started with a 2-back block and comprised 8 blocks in total. From the second block on, the *n*-back level depended on performance in the previous block, i.e., the *n*-back level increased by one if both hits > 0.7 *and* correct rejections (CR) > 0.75, decreased by one if *either* hits < 0.6 *or* CR < 0.6, or both. In all other cases, the *n*-back level of the previous block remained. After the task, we measured blood pressure and the participants received orally either a placebo, 20 mg yohimbine, or 40 mg yohimbine. After a latency of 45 min, blood pressure was measured again and the participants performed a manipulation of controllability. In this task, after a fixation cross, a frame was presented on the screen that signaled either risk or safety (counterbalanced across participants). This was followed by an arrow appearing within the frame, which signaled to participants that they were to press one out of four possible buttons (left, right, lower, and upper arrow keys). Depending on the behavioral response and experimental group, the participants could then receive a brief (100 ms) electric shock. Participants in controllability groups could avoid electric shocks in risk trials by pressing the upper arrow key, irrespective of the direction the arrow pointed to. In order to keep the number and timing of shocks constant between controllability and uncontrollability groups, participants in uncontrollability groups were yoked to a participant in the controllability condition and, thus, received a shock whenever their yoked counterpart had received a shock. Participants in no-shock groups did not receive any shocks. The manipulation of controllability task was followed by another blood pressure measurement and another session of the adaptive *n*-back task as described above. We further measured blood pressure after the post-manipulation session of the adaptive *n*-back task and at the end of the experiment

Thus, the aims of the present experiment were twofold. First, we aimed to assess dose-dependent effects of noradrenergic stimulation on working memory in healthy humans. Second, we aimed to test whether noradrenaline modulates working memory performance after exposure to controllable vs. uncontrollable aversive events. For this purpose, participants first performed a baseline session of an adaptive working memory task, which allowed us to account for interindividual differences in working memory capacity, before they received orally either a placebo or a high or low dose of the alpha-2 adrenoceptor antagonist yohimbine that leads to increased noradrenergic stimulation. After a short break, a manipulation of controllability took place during which some participants could learn to avoid electric shocks (controllability groups), whereas others received the same number of electric shocks but had no instrumental control over shock delivery (yoked uncontrollability groups). We further included a no-shock control group that did not receive any shocks. Shortly after this manipulation of controllability and at a time when the drug action should peak, participants completed another working memory session to assess changes in performance after noradrenergic stimulation and manipulation of controllability (see Fig. 1).

## Methods

### Participants and experimental design

One-hundred and thirty right-handed, healthy individuals participated in this experiment after criteria for participation had been checked using a standardized telephone interview. Individuals who reported a life-time history of any diagnosed neurological or psychiatric disorders, medication intake within 4 weeks prior to participation, intake of hormonal contraceptives in females, smoking, self-reported drug abuse (i.e., more than 14 units of alcohol/week (equaling seven average glasses of beer or wine) or the use of any illicit drugs), a body mass index (BMI) lower than 19 or higher than 27, cardiovascular disorders, diagnosed high or low blood pressure, diagnosed diabetes, kidney disease, liver disease, or thyroid hyper- or hypofunction were excluded from participation. The sample size was based on previous studies on noradrenaline effects on cognitive functions (Kluen et al. 2017; Woodcock et al. 2019) and an a priori power calculation using the software G*Power (Faul et al. 2007) showing that this sample size—in combination with a mixed within-between design including pre and post measurements of working memory—is sufficient to detect a medium-sized interaction effect of Cohen’s *f* = 0.25 with a power of 0.95. All participants provided written informed consent before participation and received a moderate monetary compensation after participation. The study protocol was approved by the ethics committee of the Hamburg Medical Association (PV5120). Nine participants were excluded from data analysis due to drop-out (moderate to strong side effects after pharmacological pill intake: *n* = 8; other health issues: *n* = 1). These exclusions resulted in a final sample size of 121 participants (55 female, age: *M* = 24.93 (*SD* = 3.88), BMI: *M* = 22.84 (*SD* = 2.03)).

We used a double-blind, fully crossed, placebo-controlled, between-subjects design with the factors noradrenergic manipulation (40 mg yohimbine, 20 mg yohimbine, placebo) and manipulation of controllability (controllability, uncontrollability, no-shock control), resulting in nine experimental groups to which participants were randomly assigned. All testing took place between 8 a.m. and 1 p.m.

### Pharmacological manipulation

Participants received orally either a placebo, 20 mg, or 40 mg yohimbine, an alpha-2 adrenoreceptor-antagonist that has been previously shown to increase noradrenergic stimulation (Ivanov and Aston-Jones 1995; Langer 1997; Starke et al. 1989). Timing and dosages of drug administration were chosen in accordance with previous studies (Kluen et al. 2017; Schwabe et al. 2012; Woodcock et al. 2019). As an indirect manipulation check, blood pressure was measured before medication intake, 45 min after medication intake, about 70 min after medication intake (after the controllability manipulation), about 85 min after medication intake (after the second *n*-back session), and about 105 min after medication intake (at the end of the experiment; see Fig. 1).

### Working memory assessment

To assess working memory performance before and after medication intake and the manipulation of controllability, we used an adaptive *n*-back task (Jaeggi et al. 2008; Kirchner 1958), which is known to be sensitive to cognitive deficits (Goldman-Rakic 1994; Honzel et al. 2014; Snyder 2013) and stress (Qin et al. 2009). The participants were presented with a sequence of one-digit numbers on a computer screen and were asked to indicate for each number whether the number was identical to the one *n* trials before or not (“yes”/“no”) by pressing one of two buttons (left arrow, right arrow) on the keyboard (see Fig. 1). Each trial consisted of a stimulus presentation (500 ms), a response window (including stimulus presentation; 1500 ms), and an inter-trial interval (1500 ms). At the beginning of the task, the participants performed two brief training blocks involving 12 trials each (a 2-back and 3-back block). The actual task version comprised eight blocks consisting of 24 trials. While the first block was always a 2-back block, all subsequent *n*-back levels were adaptively determined based on participants’ performance in the preceding block and could thus vary between 2-back and 8-back: The *n*-back level was increased by one if both the hit rate in the preceding block was higher than 70% and the rate of correct rejections was higher than 75% (unless the level of the previous block was 8-back, which led to another 8-back block under these performance criteria). Conversely, the *n*-back level was reduced by one for the next block if the hit rate or correct rejection rate (or both) of the preceding block was below 60%, unless the level of the previous block was 2-back (in this case, the level remained at 2-back). If the hit rate was between 60 and 70% (and the correct rejection rate between 60 and 75%), the *n*-back level selected for the next block was identical to the one of the preceding block. As the adaptive nature of the task resulted in different *n*-back levels for each participant and session, we extracted an accumulated *n*-back parameter by adding up all *n*-back levels reached within each session and further extracted the highest *n*-back level reached in each session as measures of performance. After each button press made within the accepted response window, a rectangle appeared around the selected response option on the screen to ensure that participants did not confuse button-response assignments and responded within the restricted response window. Between blocks, a fixation cross was presented on the screen (2000 ms), which was followed by the announcement of the next *n*-back level (8000 ms).

After medication intake and the manipulation of controllability, participants performed a second *n*-back session, which was identical to the baseline session, except that there was no training phase.

### Manipulation of controllability

About 45 min after pill intake, participants underwent a manipulation of controllability. Importantly, this manipulation differed substantially between the controllability, uncontrollability, and no-shock control groups. While participants in the controllability group could learn a behavioral response to avoid electric shocks, participants in the uncontrollability group received the same number of electric shocks as their yoked counterpart in the controllability group, but had no instrumental control over shock administration. Participants in the no-shock control group did not receive any shocks and served as control group. The task comprised 50 risk and 50 safe trials for participants in the controllability and uncontrollability groups, whereas all trials were neutral for the no-shock control groups. Each trial started with a white fixation cross presented on a black background (6000–8000 ms), followed by a frame, which could be a circle or square and signaled risk or safety, respectively. Shortly after frame onset (500 to 1000 ms), an arrow pointing to the left or right appeared within the frame. Both frame and arrow stayed on the screen until participants pressed one out of four buttons on the keyboard or until a maximum response window of 2000 ms expired. Then, a blank screen was shown on the screen (5000 to 7000 ms), which could be followed by a brief electric shock (100 ms) depending on the experimental group and behavioral response given (see Fig. 1). Participants of all groups were instructed to press one out of four buttons on the keyboard whenever an arrow appeared on the screen. Participants in the controllability groups could learn to avoid electric shocks in risk trials by pressing the top arrow key on the keyboard while risk frame and arrow were shown on the screen. Thus, participants in the controllability groups received shocks in risk trials in which they gave a wrong or no behavioral response. Participants in the uncontrollability groups were yoked to a participant in the controllability groups, i.e., we replayed the exact same sequence of trials and shock deliveries of the participant in the controllability group for the respective yoked participant in the uncontrollability group. Consequently, participants in the controllability and uncontrollability groups received the same number of shocks at the exact same timings, the only difference being that there was no contingency between behavioral responses and shock administration for participants in the yoked uncontrollability group. Accordingly, participants in the uncontrollability group received an electric shock whenever their yoked counterpart in the controllability group had received a shock. As a fully deterministic shock administration in the controllability group would have resulted in a sudden and clear-cut end of electric shocks in both the controllability and yoked uncontrollability group, bearing the risk of an illusory control in the yoked uncontrollability group, participants in the controllability group additionally received an electric shock in 10% of risk trials irrespective of their behavioral response. To prevent that this would affect their perception of controllability, participants in the controllability group were instructed that they could “significantly decrease the risk of receiving a shock when performing the correct response,” whereas participants in the uncontrollability group were instructed that they could “reliably avoid electric shocks when performing the correct response.” Furthermore, to ensure differentiation between risk and safe trials from the beginning of the task on, the first three risk trials were always followed by a shock. The trial order was pseudo-randomized to avoid a series of more than three trials of the same type (risk vs. safe) and frame-trial type associations were counterbalanced across participants.

### Experimental procedure

After providing written informed consent, participants performed the baseline session of the adaptive *n*-back task (about 12 min) and completed a brief rating questionnaire about the task. We then selected individual shock intensity levels for the manipulation of controllability. To this end, we placed shock electrodes to participants left lower leg and selected a shock intensity that was rated to be “unpleasant, but not yet painful.” We further asked the participants to rate the unpleasantness of the selected shock intensity on a scale from 0 to 100 and measured blood pressure before we administered the pharmacological treatment. During the waiting period after pill intake, the participants completed the German versions of the State-Trait Anxiety Inventory (STAI; Spielberger et al. 1970), Beck’s Depression Inventory (BDI; Beck and Steer 1987), Trier Inventory of Chronic Stress (TICS; Schulz et al. 2004), and the Attributional Style Questionnaire for adults (ASF-E; Poppe et al. 2005) to control for potential group differences in state and trait anxiety, depressive mood, chronic stress level, and attributional style. Forty-five minutes after pill intake, we measured blood pressure, briefly checked with participants whether the previously selected shock intensity was still tolerable and adjusted the intensity if necessary. The participants then underwent the manipulation of controllability, which lasted for about 25 min. After the completion of this manipulation task, we measured blood pressure, removed shock electrodes, and asked participants again to rate the unpleasantness of shocks during the experiment on a scale from 0 to 100. In order to assess the subjective experience of the task, the participants then rated their experienced level of controllability, helplessness, stress, and motivation on a scale from 0 to 100. The participants then completed the second session of the adaptive *n*-back task, which was followed by a measurement of blood pressure and another rating questionnaire about the task. At the end of the experiment, the participants in the uncontrollability group were debriefed that they had received deceptive instructions about the controllability of electric shocks and all participants received a moderate monetary compensation for participation.

### Data analysis

In line with earlier studies that used an adaptive *n*-back task (Jaeggi et al. 2008; Jaeggi et al. 2011), we chose individual *n*-back levels that were reached in a session as a dependent measure of working memory performance. More specifically, we calculated an accumulated parameter of *n*-back levels reached in each *n*-back session by adding up all the *n*-back levels reached in the respective session. As the task started with a 2-back block for each participant, which did therefore provide no information about performance, the level of the first block (2-back) was not added to the accumulated parameter, and instead, we added the hypothetical level that would have followed the last block of the session. Thus, the accumulated *n*-back level could vary between 16 and 49. In addition to the accumulated *n*-back level, we further analyzed the highest *n*-back level reached in each session (range: 2 to 8). We then assessed changes in working memory performance using repeated-measures analyses of variance (ANOVAs) with session (baseline vs. post-treatment) as a within-subject factor and noradrenergic manipulation (40 mg yohimbine vs. 20 mg yohimbine vs. placebo) and manipulation of controllability (controllability vs. uncontrollability vs. no-shock control) as between-subject factors. We further complemented the results of our frequentist analyses by additional Bayesian repeated-measures ANOVAs in order to explicitly test for evidence in favor of the null hypothesis. Additional analyses were performed to take into account findings from a previous study showing that objective and subjectively experienced controllability may substantially differ and that subjective perceptions of control rather than objective group assignments affect working memory performance. More specifically, a recent study from our lab showed that low subjective uncontrollability was associated with reduced working memory performance, whereas no differences in performance were observed between groups based on objective (un)controllability (Wanke and Schwabe 2020). Thus, we additionally performed all analyses with the factor subjective controllability instead of experimental controllability. To this end, we performed a median split based on controllability ratings in the experimental controllability and uncontrollability groups and assigned participants below the overall median (Med = 80) into a subjective low controllability group and participants above the median into a subjective high controllability group. Participants in the no-shock control group also served as the control group in analyses involving the factor subjective controllability. Data analysis was performed using SPSS 22 (IBM, NY, USA) and JASP (version 0.8.6.0; JASP Team 2018), and all reported *p* values are two-tailed. We performed a range of tests and acknowledge the need to control for multiple testing in general. However, as we report negative findings, we consider it more appropriate to refrain from corrections for multiple testing, as in this case, the absence from correction procedures is even more conservative.

## Results

### Manipulation check: yohimbine increases systolic and diastolic blood pressure

**Fig. 2:**
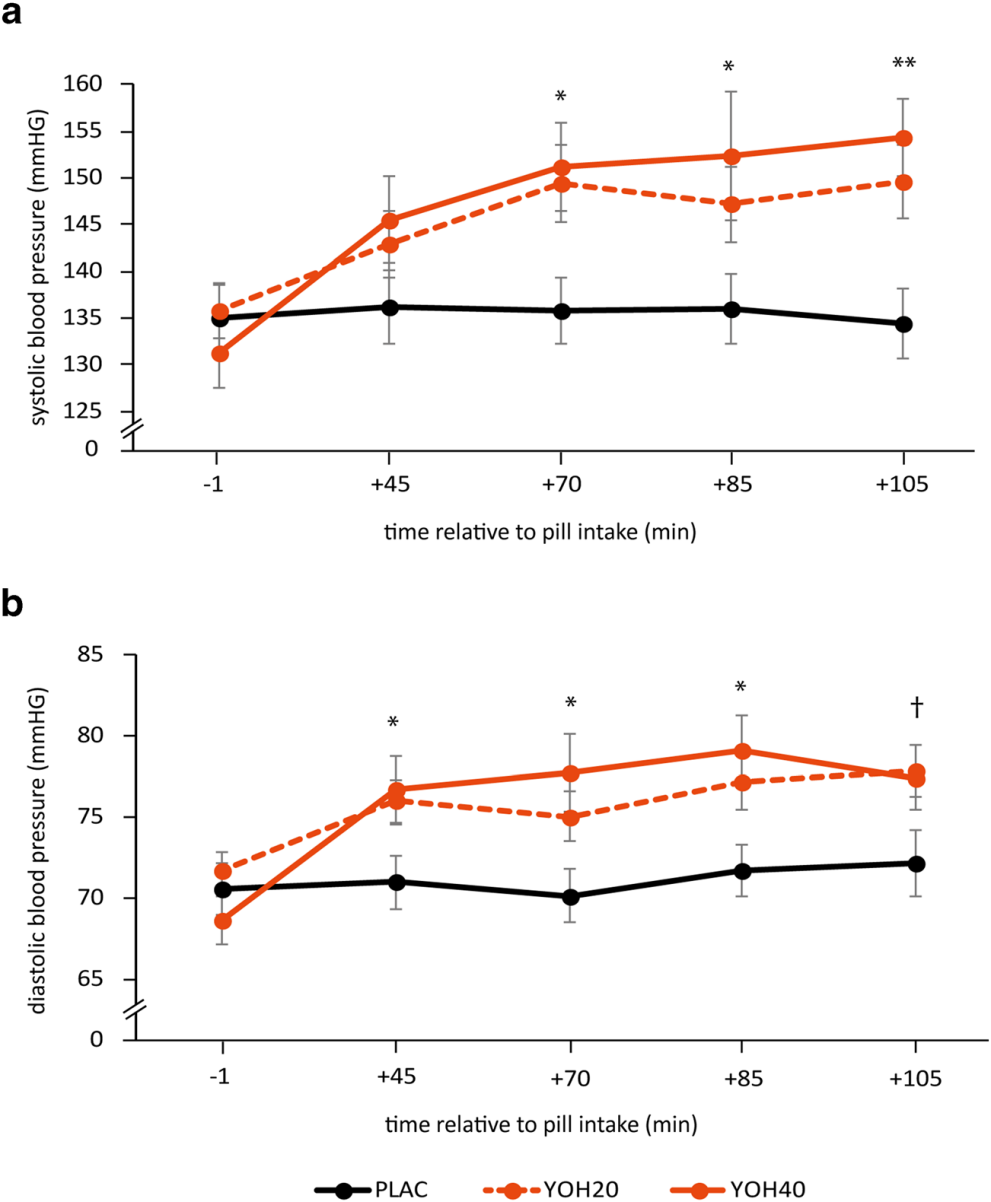
Physiological responses to pharmacological treatment. Mean **a** systolic and **b** diastolic blood pressure before, as well as 45, 70, 85, and 105 min after pharmacological pill intake. Asterisks indicate significant differences between yohimbine and placebo groups (^†^*p* < 0.1, *p < 0.05, **p < 0.01). Error bars represent standard errors of the mean. *mmHg*, millimeters of mercury; *PLAC*, placebo; *YOH20*, 20 mg yohimbine; *YOH40*, 40 mg yohimbine

Yohimbine was associated with significant increases in systolic and diastolic blood pressure (see Fig. 2), which are often considered to be indirect measures of noradrenergic arousal (Goldberg et al. 1983; Swann et al. 2005). As expected, there was a significant interaction between noradrenergic manipulation (40 mg yohimbine, 20 mg yohimbine, placebo) and time point of measurement for both systolic (*F*(8,464) = 5.370, *p* < 0.001, *η*^2^ = 0.085) and diastolic blood pressure (*F*(8,464) = 3.608, *p* < 0.001, *η*^2^ = 0.059), indicating that blood pressure changed over time depending on the pharmacological manipulation. Before the noradrenergic manipulation, there were no differences in systolic and diastolic blood pressure between pharmacological groups (systolic: *F*(2,118) = 0.496, *p* = 0.610, *η*^2^ = 0.008; diastolic: *F*(2,118) = 1.043, *p* = 0.356, *η*^2^ = 0.017). After the manipulation, diastolic blood pressure was significantly higher in the yohimbine groups than in the placebo groups 45 min after pill intake (40 mg: *t*(76) = − 2.184, *p* = 0.032; 20 mg: *t*(86) = − 2.395, *p* = 0.019), and both systolic and diastolic blood pressure were significantly elevated in the yohimbine groups 70 min (40 mg: systolic: *t*(76) = − 2.616, *p* = 0.011; diastolic: *t*(76) = − 2.709, *p* = 0.008; 20 mg: systolic: *t*(86) = − 4.490, *p* = 0.015; diastolic: *t*(86) = − 2.176, *p* = 0.032), 85 min (40 mg: systolic: *t*(86) = − 2.225, *p* = 0.029; diastolic: *t*(76) = − 2.839, *p* = 0.006; 20 mg: systolic: *t*(86) = − 2.005, *p* = 0.048; diastolic: *t*(86) = − 2.354, *p* = 0.021), and 105 min after the pharmacological treatment (40 mg: systolic: *t*(74) = − 3.534, *p* = 0.001; diastolic: *t*(74) = − 1.752, *p* = 0.084; 20 mg: systolic: *t*(86) = − 2.809, *p* = 0.006; diastolic: *t*(86) = −2.141, *p* = 0.035). Between the 20 and 40 mg yohimbine groups, there was a significant difference in increase between baseline blood pressure and blood pressure 45 min after pill intake (systolic: *F*(1,74) = 5.220, *p* = 0.025, *η*^2^ = 0.066; diastolic: *F*(1,74) = 5.247, *p* = 0.025, *η*^2^ = 0.066), and further between systolic blood pressure at baseline and at the end of the experiment (systolic: *F*(1,72) = 4.623, *p* = 0.035, *η*^2^ = 0.060; diastolic: *F*(1,72) = 1.336, *p* = 0.252, *η*^2^ = 0.018). Neither systolic nor diastolic blood pressure was affected by the manipulation of controllability, as indicated by the lack of interaction between manipulation of controllability and time points of measurement for both systolic (*F*(8,464) = 1.245, *p* = 0.271, *η*^2^ = 0.021) and diastolic (*F*(8,464) = 1.461, *p* = 0.169, *η*^2^ = 0.025) blood pressure.

### Successful learning of the instrumental response to avoid electric shocks during the manipulation of controllability

During the manipulation of controllability, participants in the controllability and uncontrollability groups received on average 18.77 (*SD* = 10.14) shocks. Due to slightly imperfect yoking (experimenter error: *n* = 1; exclusion due to sickness: *n* = 2), the number of shocks varied minimally between experimental controllability and uncontrollability groups (controllability groups: *M* = 18.63 (*SD* = 10.42); uncontrollability groups: *M* = 18.93 (*SD* = 9.99)), but this difference was not significant (*t*(78) = 0.131, *p* = 0.896). There was further no difference in shock intensity levels between controllability and uncontrollability groups (*t*(77) = −0.360, *p* = 0.720; *M*_con_ = 54.29 V (*SD* = 12.18); *M*_uncon_ = 55.26 V (*SD* = 11.69)) or between pharmacological groups (*F*(2,76) = 0.714, *p* = 0.493, *η*^2^ = 0.018; *M*_PLAC_ = 56.40 V (*SD* = 11.00); *M*_YOH20_ = 52.70 V (*SD* = 13.94); *M*_YOH40_ = 55.25 V (*SD* = 10.15)); and there were also no differences between controllability and uncontrollability groups in pain ratings (*t*(61.262) = − 1.074, *p* = 0.287).

**Fig. 3:**
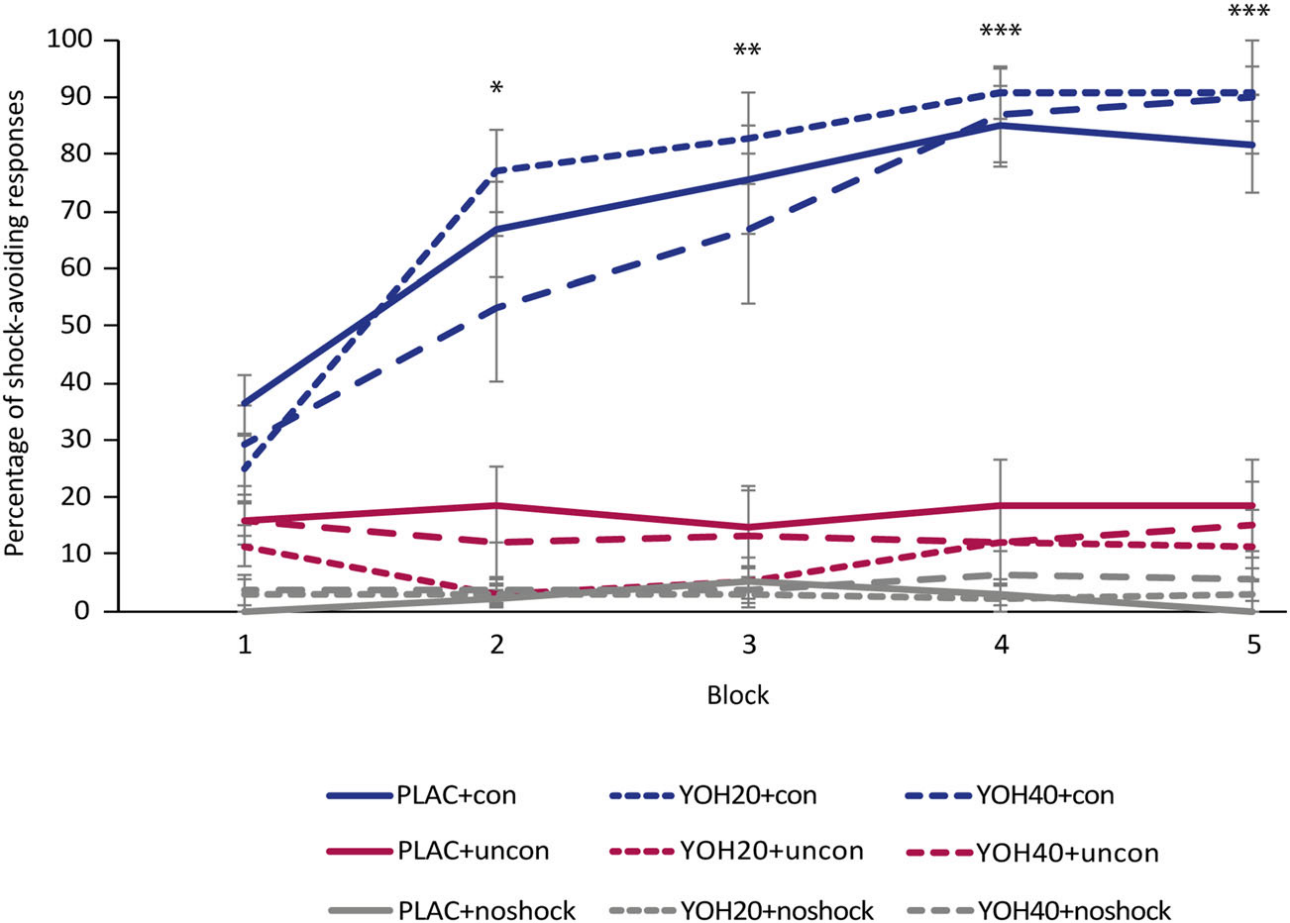
Learning of instrumental shock-avoiding response. Participants in controllability groups show a significant increase in performing the instrumental response required to avoid electric shocks. As expected, participants in uncontrollability and no-shock control groups do not display an increase in performing responses that avoid shocks in the controllability condition. Blocks comprise 10 risk trials each. Error bars represent standard errors of the mean. *p < 0.05, **p < 0.01, ***p < 0.001. *PLAC*, placebo; *YOH20*, 20 mg yohimbine; *YOH40*, 40 mg yohimbine; *con*, controllability; *uncon*, uncontrollability

Importantly, participants in the controllability groups successfully learned how to avoid shocks, as shown by a significant increase in performing the correct instrumental shock-avoiding response in risk trials over the course of the experiment (*F*(4,160) = 66.291, *p* < 0.001, *η*^2^ = 0.624), whereas the yoked uncontrollability groups and no-shock control groups did not show an increase in performing the response that avoided shock delivery in the controllability group (trial type (safe vs. threat) × time (block 1 vs. block 2 vs. block 3 vs. block 4 vs. block 5) × manipulation of controllability (controllability vs. uncontrollability vs. no-shock) interaction (*F*(8,468) = 13.921, *p* < 0.001, *η*^2^ = 0.192; see Fig. 3). Next, we investigated whether our manipulation of controllability affected subjective perceptions of controllability. As expected, we observed higher subjective ratings of controllability in the experimental controllability groups (*M* = 80.36, *SD* = 21.11) than in the experimental uncontrollability groups (*M* = 61.75, *SD* = 31.74; *t*(67.408) = 3.110, *p* = 0.003).

We then assessed whether the noradrenergic manipulation affected the behavioral responses during the manipulation of controllability. Therefore, we first compared whether learning performance in the controllability groups, expressed as number of shocks received, differed depending on the pharmacological manipulation. This analysis showed that the noradrenergic manipulation had no effect on learning performance of the controllability groups (*F*(2,37) = 0.583, *p* = 0.563, *η*^2^ = 0.031). Next, we assessed the effects of the pharmacological manipulation on subjective controllability measures. These analyses showed that the noradrenergic manipulation did not affect subjective controllability ratings in the experimental controllability and uncontrollability groups (main effect of noradrenergic manipulation: *F*(2,76) = 0.746; *p* = 0.478, *η*^2^ = 0.019; interaction noradrenergic manipulation × manipulation of controllability: *F*(4,76) = 0.034, *p* = 0.967, *η*^2^ = 0.001). The pharmacological manipulation did not further affect participants’ pain intensity ratings (*F*(2,75) = 2.150, *p* = 0.124, *η*^2^ = 0.054) but did interact with manipulation of controllability (*F*(2,75) = 3.501, *p* = 0.035, *η*^2^ = 0.085). Follow-up *t* tests for each of the pharmacological groups showed that this interaction was driven by significantly higher pain ratings in the uncontrollability placebo group as compared with the controllability placebo group after the manipulation (*t*(21.195) = − 2.489, *p* = 0.021; *M*_PLAC+con_ = 53.00 (*SD* = 24.33); *M*_PLAC+uncon_= 70.67 (*SD* = 12.80), but this difference did not survive a Bonferroni-corrected significance threshold of *p* = 0.017.

### Working memory is not affected by noradrenergic stimulation

**Fig. 4:**
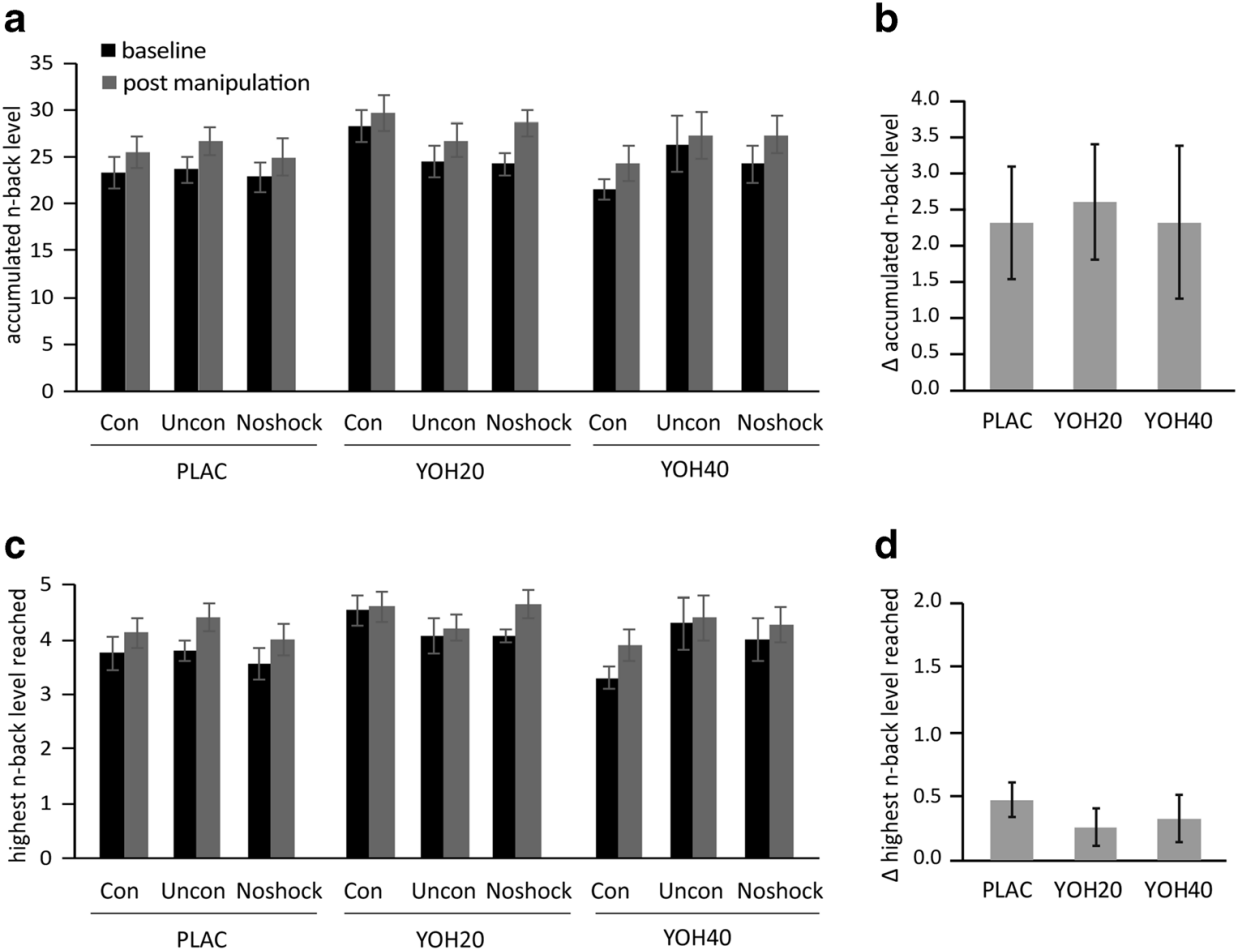
Performance in the adaptive *n*-back task before and after the pharmacological manipulation and manipulation of controllability. Neither **a**, **b** the accumulated *n*-back level nor **c**, **d** the highest *n*-back level reached was affected by noradrenergic stimulation or the manipulation of controllability, and there was no significant interaction between noradrenergic stimulation and manipulation of controllability. Error bars represent standard errors of the mean. *PLAC*, placebo; *YOH20*, 20 mg yohimbine; *YOH40*, 40 mg yohimbine; *con*, controllability; *uncon*, uncontrollability

We then assessed whether the noradrenergic manipulation and manipulation of controllability affected the working memory performance. To this end, we calculated the accumulated *n*-back level during the baseline and post-manipulation sessions in order to determine changes in working memory performance within participants and between experimental groups. As an additional performance measure, we used the highest *n*-back achieved during each session. During the baseline *n*-back session, participants achieved an average accumulated *n*-back level of *M* = 24.388 (*SD* = 6.296; range: 16–46), while the average of the highest *n*-back level was *M* = 3.942 (*SD* = 1.090; range: 2–7). Baseline working memory performance was comparable in the experimental groups, with respect to both the accumulated *n*-back baseline level (main effect of noradrenergic manipulation: *F*(2,112) = 1.682, *p* = 0.191, *η*^2^ = 0.029; main effect of manipulation of controllability: *F*(2,112) = 0.239, *p* = 0.787, *η*^2^ = 0.004; noradrenergic manipulation × manipulation of controllability interaction: *F*(4,112) = 1.399, *p* = 0.239, *η*^2^ = 0.048) and the highest *n*-back level achieved (main effect of noradrenergic manipulation: *F*(2,112) = 2.625, *p* = 0.077, *η*^2^ = 0.045; main effect of manipulation of controllability: *F*(2,112) = 0.316, *p* = 0.730, *η*^2^ = 0.006; noradrenergic manipulation × manipulation of controllability interaction: *F*(4,112) = 1.469, *p* = 0.216, *η*^*2*^ = 0.050). We then assessed whether the change in performance between baseline and post-manipulation performance was associated with the pharmacological manipulation of noradrenergic activity and the (objective) controllability over aversive events. To this end, we performed a repeated-measures ANOVA on the accumulated *n*-back level involving the within-subject factor session (baseline vs. post-treatment) and the between-subject factors noradrenergic manipulation (40 mg yohimbine vs. 20 mg yohimbine vs. placebo) and manipulation of controllability (controllability vs. uncontrollability vs. no-shock). We performed identical analyses using the highest *n*-back level as the dependent variable. In line with previous studies (Bogdanov and Schwabe 2016; Wanke and Schwabe 2020), we observed a significant training effect in working memory performance, as shown by significant increases in the accumulated *n*-back level (*F*(1,112) = 23.469, *p* < 0.001, *η*^2^ = 0.173; *M*_pre_ = 24.388; *M*_post_ = 26.802) and highest *n*-back level (*F*(1,112) = 13.263, *p* < 0.001, *η*^2^ = 0.106; *M*_pre_ = 3.942; *M*_post_ = 4.273) from baseline to post-manipulation. These analyses did, however, not reveal any significant interaction effects of session and noradrenergic manipulation, session and controllability manipulation, or session, noradrenergic manipulation, and controllability manipulation on working memory performance, as indicated by a lack of significant interaction effects for the accumulated *n*-back level (all *F*s *≤* 0.381, *p*s *≥* 0.392, *η*^2^s *≤* 0.017) and the highest *n*-back level (all *F*s *≤* 0.702, *p*s *≥* 0.498, *η*^2^s *≤* 0.016; see Fig. 4).

### Complementary Bayesian analyses provide strong evidence for a lack of effect of noradrenergic stimulation on working memory performance

As a lack of significant findings in classical frequentist analyses does not allow to draw conclusions about the (non-)existence of effects (Dienes et al. 2018; Hoijtink et al. 2019; Lee and Wagenmakers 2014; Wagenmakers et al. 2018), we next performed Bayesian repeated-measures ANOVAs using JASP (version 0.8.6.0; JASP Team 2018) to investigate whether there is Bayesian evidence for a lack of a relationship between working memory performance and noradrenergic stimulation and/or (un)controllability. We thus entered the accumulated *n*-back level and the highest *n*-back level at baseline and post-treatment, respectively, into a repeated-measures Bayesian ANOVA with the between-subject factors noradrenergic manipulation and manipulation of controllability. To assess whether there is Bayesian evidence in favor of the absence of an association between working memory and noradrenergic activity as well as (objective) controllability over aversive events, we analyzed Bayes Factors comparing the likelihood of the alternative hypothesis against the likelihood of the null hypothesis (*BF*_10_), i.e., the non-existence of an effect. More specifically, we calculated posterior inclusion probabilities (*BF*_inclusion_) using the effects across matched models approach suggested by Mathôt (2017), allowing to assess the effects of interaction terms under exclusion of evidence for respective main effects. We employed conventional criteria (Lee and Wagenmakers 2014; Quintana and Williams 2018) to interpret Bayes Factors, i.e., we classified a *BF* between 0.1 and 0.33 as moderate evidence and a *BF* between 0.033 and 0.1 as strong evidence in favor of the null hypothesis. For our main dependent variable, the change in accumulated *n*-back level from baseline to post-manipulation, we observed strong evidence in favor of a lack of noradrenergic effects on working memory (session × noradrenergic manipulation: *BF*_inclusion_ = 0.086). For all other effects investigated, namely interactions between *n*-back session and manipulation of controllability, and an interaction of *n*-back session manipulation of controllability and noradrenergic manipulation, our analyses provided moderate evidence for the null hypothesis (session × manipulation of controllability: *BF*_inclusion_ = 0.178; interaction session × noradrenergic manipulation × manipulation of controllability: *BF*_inclusion_ = 0.142). Analyses based on the highest *n*-back level revealed the same pattern of results and provided further moderate evidence for a lack of effects (session × noradrenergic manipulation: *BF*_inclusion_ = 0.142; session × manipulation of controllability: *BF*_inclusion_ = 0.110; session × noradrenergic manipulation × manipulation of controllability: *BF*_inclusion_ = 0.133).

### Additional (iterative) analyses based on subjective controllability ratings

Based on previous findings indicating that subjectively perceived controllability rather than objective controllability affects cognitive functioning (Wanke and Schwabe 2020), we additionally performed all analyses as described above, but replaced the factor manipulation of controllability by subjective controllability (high subjective controllability, low subjective controllability, no-shock). In line with the previous results, we did not observe significant interaction effects for accumulated *n*-back level (all *F*s *≤* 0.657, *p*s *≥* 0.593, *η*^2^s *≤* 0.024). An identical pattern of results was observed for all analyses based on the highest *n*-back level as dependent variable (all *F*s *≤* 0.588, *p*s *≥* 0.557, *η*^2^s *≤* 0.022).

In order to evaluate evidence for a relationship between working memory performance and noradrenaline and subjective controllability, we then performed Bayesian ANOVAs. In line with previous analyses, a Bayesian ANOVA based on accumulated *n*-back level provided strong evidence for a lack of noradrenergic effects on accumulated working memory performance (session × noradrenergic manipulation: *BF*_inclusion_ = 0.090), and we observed further moderate evidence for a lack of relationship between subjective controllability and accumulated working memory performance (session × subjective controllability: *BF*_inclusion_ = 0.146) and an interaction between session, noradrenergic manipulation, and subjective controllability (*BF*_inclusion_ = 0.177). A further analysis based on the highest *n*-back level yielded a similar pattern of results and provided moderate evidence to discard a noradrenaline effect on highest *n*-back level (session × noradrenergic manipulation: *BF*_inclusion_ = 0.155), and to further discard an effect of subjective controllability on highest *n*-back level (session × subjective controllability*: BF*_inclusion_ = 0.151) and an interaction between session, noradrenaline, and subjective controllability (*BF*_inclusion_ = 0.185).

## Discussion

It is commonly assumed that noradrenergic arousal interferes with prefrontal functions, including working memory (Arnsten 2009). However, evidence in support of this idea comes mainly from studies in non-human animals (Arnsten 2011; Chamberlain and Robbins 2013). Human data on the role of noradrenaline in working memory are scarce and the existing findings are inconsistent (Chamberlain et al. 2006; Chamberlain and Robbins 2013). In the present dose-dependent study, we investigated whether low or high dosages of the alpha-2 adrenoceptor antagonist yohimbine affected the working memory performance in healthy participants, while controlling for individual differences in working memory capacity. Our findings show that increased noradrenergic stimulation through yohimbine did not affect the working memory performance. Bayesian analyses provided further direct evidence for the absence of yohimbine effects on working memory. Moreover, yohimbine did not affect the acquisition of instrumental control over aversive events, the subjective sense of helplessness after exposure to uncontrollable aversive events, or the relation between uncontrollability and working memory performance.

The present experiment provides evidence for the absence of a relationship between noradrenergic stimulation and working memory performance. Importantly, the use of an adaptive version of the *n*-back task allowed us to rule out ceiling (or floor) effects in performance as a potential explanation for the lack of an effect. The experimental design used in the present study further accounted for interindividual differences in working memory performance due to a baseline session before the pharmacological treatment and manipulation of controllability took place. As differences in dosage may be another potential explanation for inconclusive findings in humans, we used both a high and low dosage of yohimbine, the effectiveness of the which was indicated by blood pressure changes, but did not find changes in working memory performance after either 20 mg or 40 mg of yohimbine. While earlier studies in humans did not find a clear relationship between alpha adrenergic receptors and working memory performance (Chamberlain and Robbins 2013), this result is in stark contrast to previous findings in non-human primates (Arnsten 2011). These divergent findings may be explained by the use of different tasks targeting working memory. Comparisons between widely used working memory tasks have shown that the tasks are not pure measures of working memory but also tap into other cognitive domains, such as information processing speed (Miller et al. 2009), and that some tasks are not or only weakly correlated (Kane et al. 2007; Miller et al. 2009). Thus, noradrenaline might disrupt only specific aspects of working memory tasks, and thus only impair performance on tasks that incorporate a specific feature, for instance those incorporating a visuo-spatial component (Ellis and Nathan 2001). The idea of a potential distinction between noradrenergic effects on spatial and non-spatial working memory tasks is in line with the observation that a range of studies that found an association between noradrenergic stimulation and working memory performance indeed used spatial working memory tasks (Coull et al. 1995; Ellis and Nathan 2001) and that research in non-human primates used delayed matching-to-sample tasks that depend on visuo-spatial properties (Birnbaum et al. 1999; Franowicz et al. 2002).

Alternatively, the lack of an association between noradrenergic stimulation and working memory performance in the present study and inconsistent findings in earlier studies (Chamberlain et al. 2006; Chamberlain and Robbins 2013) may be due to differences between species. Although brains of humans and non-human primates are thought to be similar in structure and function, there is evidence for important differences between species that concern mainly the prefrontal cortex (Smaers et al. 2017; Teffer and Semendeferi 2012). Beyond changes in the distribution of different cell types, and the addition of novel cortical areas that do not have an analogue in the non-human brain, it is suggested that a phylogenetically recent reorganization of the human frontal cortex resulted in a distributed neural brain network with a dominant role of the prefrontal cortex (Smaers et al. 2017), thus causing differential processing of information in the human and non-human brain. Another potential explanation for the inconsistent findings regarding the relationship between noradrenaline and working memory in humans may include the variety of (noradrenergic) receptors and potential pharmacological mechanisms. The pharmacological agent used in the present study (i.e., yohimbine) affects primarily alpha-2 adrenergic receptors but is known to interact to a lesser extent with alpha-1 adrenergic receptors, and also with dopaminergic and serotonergic receptors in high concentrations (Dukes 1988). Thus, the assessment of dose-dependent yohimbine effects may be limited due to potential interaction with other receptors exerting opposite effects on cognitive functions (Ellis and Nathan 2001). It further cannot be ruled out that adrenergic receptors other than the alpha-2 type mediate noradrenergic effects on working memory in humans or that the balance between receptor type and noradrenergic stimulation drives potential (lack of) effects.

Based on initial evidence in rodents suggesting that noradrenaline may be involved in the control of aversive events (Weiss et al. 1981), we tested, in addition to changes in working memory, the impact of yohimbine on the instrumental control of aversive events and (un)controllability-related changes in working memory. Our results showed that yohimbine did not impair participants’ ability to learn how to exert instrumental control over aversive events and did further not affect subjectively perceived control. Furthermore, in contrast to previous research in rodents that observed a depletion of noradrenaline in the locus coeruleus, leading to increased noradrenergic activity, and escape deficits after exposure to uncontrollable as opposed to controllable aversive events (Weiss et al. 1981; Weiss and Simson 1988), we found that noradrenergic stimulation through yohimbine did not modulate controllability-dependent effects on working memory. This finding is somewhat consistent with more recent studies in rodents that have pointed to a role of serotonin in cognitive deficits after exposure to uncontrollable aversive events (Amat et al. 2005; 2006; 2008).

Somewhat surprisingly, we did also not observe an effect of either experimental or subjective uncontrollability on working memory performance, although the latter finding needs to be interpreted with caution due to only moderate Bayes factors. This result is inconsistent with an earlier study in which we obtained working memory deficits after subjectively experienced uncontrollability (Wanke and Schwabe 2020). Reasons for this discrepancy may stem from differences in the experimental setup between the present and the previous study. First, in the present study, we used an adaptive version of the *n*-back task, whereas the previous study employed fixed *n*-back levels. While the adaptive task may have enabled the quantification of large interindividual differences in working memory capacity at baseline, the adaptive nature of the task may have not been cognitively challenging enough to detect subtle changes in individual performance after the manipulation of controllability as the level of difficulty depended directly on individuals’ performance. Furthermore, as the pharmacological drug required some time to reach effectiveness, there was a significantly longer latency between baseline and post-manipulation session in the present study, which may have caused fatigue and thus abolished effects of subjective (un)controllability. Another potential explanation for the lack of effect might be the presence of the pharmacological treatment in the present study. Pharmacological pill intake and consideration of potential side effects might in itself constitute a source of stress and might have taken focus from electric shocks as actual stressors, thus diminishing the impact of perceived control over electric shocks on subsequent cognitive processes. Lastly, in our previous study, working memory testing took place in an MRI scanner, which may have further resulted in increased levels of arousal during working memory assessments.

Finally, one might argue that the rather complex design of the present experiment, including nine experimental groups, could have prevented the detection of significant effects. We consider this alternative rather unlikely. First, we used a pre-post design that allowed us to take individual differences in working memory into account. Furthermore, the measurement of working before and after the experimental manipulation increased the statistical power significantly. In fact, the pre-post measurements of working memory, in combination with the rather large sample size, resulted in a statistical power of 0.95 for the interaction effects of yohimbine and instrumental control, which was even higher for the main effect of yohimbine. Thus, we consider the chosen design as an explanation for the absence of a significant effect of yohimbine on working memory rather unlikely.

In sum, the present dose-dependent study suggests evidence that increased noradrenergic stimulation through alpha-2 adrenoceptor antagonism does not interfere with (non-spatial) working memory performance in healthy humans. Our results further suggest that noradrenergic stimulation does not affect the acquisition of instrumental control over aversive events or the level of subjective helplessness after exposure to uncontrollable stress. It should be noted that we tested the effects of noradrenergic stimulation on working memory performance in young and healthy participants. Whether elderly individuals or those with specific medical conditions are more vulnerable to noradrenaline effects on working memory remains to be tested. Moreover, as effects of yohimbine are restricted to alpha-2 adrenergic receptors, the present study cannot rule out that noradrenaline signaling through other adrenergic receptors may affect working memory performance. However, the present findings demonstrate that noradrenergic stimulation does not necessarily interfere with working memory performance, which may have relevant implications for our understanding of arousal-related mental disorders as well as for potential treatment approaches for these conditions.
